# Granulocyte colony-stimulating factor attenuates monocrotaline-induced pulmonary hypertension by upregulating endothelial progenitor cells via the nitric oxide system

**DOI:** 10.3892/etm.2013.1328

**Published:** 2013-10-07

**Authors:** JUN-FENG LIU, ZHONG-DONG DU, ZHI CHEN, ZHONG-CHAO HAN, ZHI-XU HE

**Affiliations:** 1 Laboratory of Tissue Engineering and Stem Cells, Guiyang Medical College, Guiyang, Guizhou 550004, P.R. China; 2 Department of Pediatrics, The General Hospital of Huabei Oil Field Company, Renqiu, Hebei 062552, P.R. China; 3 Department of Cardiology, Beijing Children’s Hospital, Capital Medical University, Beijing 100045, P.R. China; 4 National Engineering Research Center of Cell Products, AmCellGene Co. Ltd, Tianjin 300457, P.R. China

**Keywords:** pulmonary hypertension, model, endothelial progenitor cells, nitric oxide, granulocyte colony-stimulating factor, hemodynamics

## Abstract

Granulocyte colony-stimulating factor (G-CSF) has exhibited efficacy at preventing the progression of pulmonary hypertension (PH); however, the exact mechanism is not completely clear. The aim of the present study was to assess whether this protective effect was mediated by the upregulation of circulating endothelial progenitor cells (EPCs) via the nitric oxide (NO) system. PH was induced in male Sprague-Dawley (SD) rats by the administration of a single subcutaneous injection of monocrotaline (MCT). The rats were treated with recombinant human G-CSF (rhG-CSF, 50 μg/kg/day) by subcutaneous injection from day five to day seven subsequent to the injection of MCT. Nω-nitro-L-arginine methyl ester (L-NAME, 4 mg/kg/day) was intragastrically administered in addition to rhG-CSF as a negative intervention. The changes in hemodynamics and histology, the number and function of circulating EPCs and the concentration of plasma NO were evaluated. With the occurrence of PH in the rat model, the number and function of circulating EPCs were demonstrated to be markedly downregulated. Moreover, a reduced plasma concentration of NO was observed, which was positively correlated with the number of circulating EPCs. Administration of rhG-CSF elevated the plasma level of NO, upregulated the number and function of circulating EPCs and effectively improved pulmonary hemodynamics and vascular reconstruction. Furthermore, the positive correlation between the levels of plasma NO and circulating EPCs was also observed in the rhG-CSF treatment group. However, the protective effect of rhG-CSF in PH was attenuated by L-NAME, which mediated the downregulation of NO and the EPCs. Thus, the present study suggests that G-CSF may attenuate the progression of MCT-induced PH by improving vascular injury repair mechanisms via the NO-mediated upregulation of EPCs.

## Introduction

The term pulmonary hypertension (PH) describes a group of lung disorders characterized by a progressive increase in pulmonary arterial pressure. The common pathological feature of PH is pulmonary vascular remodeling. Persistent high blood pressure in the pulmonary circulation results in right ventricular hypertrophy and dilatation, leading to the eventual occurrence of right heart failure. Along with the advancement in studies on PH in recent years, the importance of endothelial progenitor cells (EPCs) has been recognized among the possible factors involved in the mechanism of PH. EPCs have been considered to be pivotal in maintaining the normal structure and function of the vascular endothelium (1–3), and the dysfunction of this process may, to a certain degree, contribute to the occurrence of pulmonary vascular remodeling in PH. It has been shown that the excessive apoptosis of pulmonary artery endothelial cells in PH results in the destruction of endothelial integrity (4). Furthermore, the endothelial repair effect of EPCs is not able to be implemented effectively, due to the downregulation in EPC number and function (5,6), leading to an imbalance between the lesion and the repair of the vascular endothelium and the further damage of pulmonary arteries.

EPC transplantation has been shown to be effective at preventing the progression of PH in laboratory rats (4,7). However, this procedure is likely to be limited in clinical practice due to difficulties in obtaining sufficient EPCs from donors during the effective treatment period; therefore, a safe and convenient protocol to improve EPCs in patients with PH is preferable. As a regulator of granulocytes, granulocyte-colony stimulating factor (G-CSF) has been used for decades, and exhibits reliability in the clinic. Furthermore, the administration of G-CSF in cardiovascular disease leads to the repair of the injured vessel and myocardium by the mobilization of bone marrow EPCs and their precursors (8). It has also exhibited efficacy at preventing the progression of PH (9). However, studies of EPC number and function following the administration of G-CSF in PH are lacking, and the mechanism underlying the protective effect of G-CSF on PH has not been fully elucidated. It has been demonstrated that nitric oxide (NO), as a signaling molecule, is required for the mobilization of bone marrow EPCs (10), while reducing the apoptosis of EPCs (11), and participating in angiogenesis and vasculogenesis (12,13). It has been shown that the cardioprotective effect of G-CSF is also mediated by the NO system (14,15). Therefore, we proposed that G-CSF attenuates PH by the NO-mediated upregulation of EPCs.

In the present study, we utilized a rat model of PH, created by the subcutaneous injection of monocrotaline (MCT), and treated the rats with recombinant human G-CSF (rhG-CSF). Nω-nitro-L-arginine methyl ester (L-NAME) was used concurrently as a negative intervention. The therapeutic effect, number and function of circulating EPCs and the concentration of plasma NO were evaluated, in order to enhance the understanding of the protective mechanisms of rhG-CSF in PH.

## Materials and methods

### Animals

Male Sprague-Dawley (SD) rats, aged 8 weeks, were purchased from Capital Medical University (Beijing, China) and housed in specific pathogen-free units of the Division of Laboratory Animals at Capital Medical University. Thirty-two rats were randomly divided into four groups: the model [MCT and phosphate-buffered saline (PBS)], rhG-CSF treatment (MCT and rhG-CSF), L-NAME intervention (MCT, rhG-CSF and L-NAME) and control (PBS) groups. Each group contained eight rats. PH was induced by a single subcutaneous injection of MCT (60 mg/kg; Sigma, St. Louis, MO, USA) (16), while PBS was administered to the controls. rhG-CSF (50 μg/kg/day; Xiamen Amoytop Biotech Co., Ltd., Xiamen, China) or PBS was subcutaneously injected from day five to day seven after the injection of MCT. Furthermore, L-NAME (4 mg/kg/day; Sigma) was intragastrically administered at the same time as the rhG-CSF injection and continued to day 21. All animal studies and protocols were approved by the Institutional Animal Care and Use Committee of Capital Medical University.

### Examination of hemodynamics

At day 21, the rats were anesthetized by an intraperitoneal injection of pentobarbital (50 mg/kg). A polyethylene catheter was inserted into the right ventricle (RV) via the right external jugular vein, and another was targeted at the ascending aorta via the right carotid artery. Right ventricular systolic pressure (RVSP) and mean aortic pressure (MAoP) were recorded using a polygraph (Nihon Kohden Corporation, Tokyo, Japan).

### Numbers of EPCs in peripheral blood

Peripheral blood was collected from the right external jugular vein into EDTA-containing tubes and 100 μl was incubated with 2 μl fluorescein isothiocyanate (FITC)-conjugated goat monoclonal antibody against mouse immunoglobulin G, and 5 μl mouse monoclonal antibody against rat vascular endothelial growth factor receptor (VEGFR)-2 (Santa Cruz Biotechnology, Inc., Santa Cruz, CA, USA). Following red cell lysis and washing with PBS, the cells were incubated for a further 20 min with 5 μl allophycocyanin (APC)-conjugated mouse monoclonal antibody against rat CD45 (BD Biosciences, Franklin Lakes, NJ, USA) and 5 μl phycoerythrin (PE)-conjugated mouse monoclonal antibody against rat CD34 (BD Biosciences). The samples were subsequently centrifuged for 5 min at 300 × g and then resuspended in 500 μl PBS and evaluated using flow cytometry (BD FACSCalibur Flow Cytometer; BD Biosciences, San Jose, CA, USA). Isotype controls were run in parallel and ~100,000 events were recorded. Circulating EPCs were defined as cells positive for CD34 and VEGFR-2, but negative for CD45 (17).

### Plasma NO measurement

Peripheral blood was centrifuged for 15 min at 1,200 × g, prior to 50 μl plasma being collected into 96-well plates for the measurement of NO. The Nitric Oxide Assay kit (Beyotime Biotechnology, Haimen, China) was based on Greiss reagent. The concentration of NO was determined by spectrophotometry (490 nm), following the addition of 50 μl Greiss reagents I (1% sulfanilamide in 0.1 mol/l HCl) and II [0.1% N-(1-naphthyl-ethylenediamine dihydrochloride)].

### EPCs cultured in vitro

Mononuclear cells were isolated from peripheral blood using Ficoll density gradient centrifugation (20 min at 400 × g without brake) and suspended with endothelial cell growth medium-2 (EGM-2; Lonza Group AG, Basel, Switzerland) in 24-well culture plates pre-coated with fibronectin (Sigma), and incubated at 37°C in a humidified environment with 5% carbon dioxide (CO_2_). Unattached cells were removed by extensive washing on day three, and the culture medium was replaced every two days thereafter. Following seven days of culture, cells were incubated with 10 μg/ml DiI-labeled acetylated low-density lipoprotein (acLDL; Molecular Probes^®^, Invitrogen Life Technologies, Carlsbad, CA, USA). Four hours later, the cells were incubated with 10 μg/ml FITC-conjugated lectin from Ulex europeus agglutinin-1 (FITC-UEA-1; Sigma) for 1 h, following fixation with 4% paraformaldehyde. The cells were subsequently examined using laser scanning confocal microscopy. Differentiating EPCs were identified by double fluorescence staining, as previously described (18).

### Function of EPCs in vitro

EPCs were detached using 0.25% trypsin following seven days of culture. EPC functions, such as proliferation, adhesion and migration, were assessed as described in a previous study (18). With regard to proliferation, the cells were cultured for 24 h in 96-well plates at a density of 10^5^ cells/ml (200 μl per well), prior to 20 μl MTT (5 g/l) being added for 4 h. Following this, the media was discarded and 100 μl dimethylsulfoxide (DMSO) was added for 10 min. The absorbance was measured at a wavelength of 490 nm. For adhesion, EPCs were incubated with EGM-2 in 24-well plates pre-coated with fibronectin (5×10^4^/well) for 30 min. Subsequent to washing three times with PBS, the attached cells were counted in a high power field (HPF). To assess the migratory ability of the cells, a modified Boyden chamber (8-μm pore size) was used. EPCs were suspended in 100 μl EGM-2 without cytokines, plus 0.5% fetal bovine serum (FBS), in the upper chamber (5×10^5^/ml) and placed in a 24-well culture plate containing 600 μl EGM-2. Following 24 h of incubation, the lower membrane of the chamber was fixed with 4% paraformaldehyde. Migrated cells were counted in a HPF, subsequent to staining with 0.1% crystal violet.

### Histological examination

Lung tissues were removed from the rats subsequent to sacrifice by decapitaiton and fixed in 10% paraformaldehyde for 24 h. Following this, serial paraffin sections (5-μm) were stained with hematoxylin and eosin for light microscopy (magnification, ×400). The medial wall thickness of the pulmonary arteriole is expressed as: Wall thickness (WT, %) = [(medial thickness × 2)/external diameter] × 100 (9).

### Statistical analysis

Data are presented as the mean ± standard deviation, and were statistically analyzed using SPSS statistical software (version 13.0; SPSS, Inc., Chicago, IL, USA). Differences were compared using one-way analysis of variance (ANOVA) tests. Correlations were calculated according to Pearson’s correlation. P<0.05 was considered to indicate a statistically significant difference.

## Results

### Characteristics of the experimental rats

Approximately 10 days following the co-administration of MCT and PBS, rats exhibited shortness of breath and a reduction in locomotor activity and food intake compared with controls. In addition, the body weight of the model rats was shown to have decreased (172.3±19.6 versus 208.4±18.5 g, P<0.05). The administration of rhG-CSF resulted in a marked improvement in the status of the rats, and there were no differences in body weight between the rhG-CSF treatment group (203.7±19.7 g) and the controls (P>0.05), while only the level of locomotor activity remained slightly decreased. However, under the negative intervention of L-NAME, the body weight of the rats (170.5±18.2 g) was markedly decreased compared with that of the rhG-CSF treatment group (P<0.05).

### Changes in hemodynamics and histology

**Figure 1:**
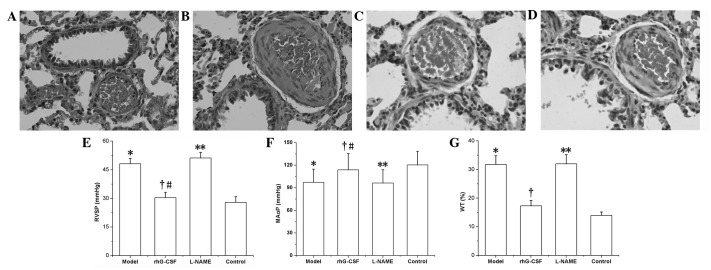
Effect of recombinant human granulocyte colony-stimulating factor (rhG-CSF) on pulmonary hypertension (PH). (A–D) Optical photomicrographs of lung stained with hematoxylin and eosin (magnification, ×400). (A) Control group, (B) model group, (C) rhG-CSF treatment group and (D) Nω-nitro-L-arginine methyl ester (L-NAME) intervention group. (E–G) Changes in (E) right ventricular systolic pressure (RVSP), (F) mean aortic pressure (MAoP) and (G) wall thickness (WT) in each group. Data are presented as the mean ± standard deviation, n=8. ^*^P<0.01 compared with the control group; ^†^P<0.01 compared with the model group; ^#^P>0.05 compared with the control group; ^**^P<0.01 compared with the rhG-CSF treatment group.

Twenty-one days following the injection of MCT, the RVSP of the rats in the model group was increased compared with that of the controls (48.13±2.85 versus 27.88±3.04 mmHg, P<0.01), while the MAoP was decreased from 120.33±18.25 mmHg in the control group to 97.24±17.52 mmHg in the model group. This indicated the occurrence of PH. The administration of rhG-CSF led to the RVSP of the rats being decreased significantly compared with that of the model group (30.38±2.83 versus 48.13±2.85 mmHg, P<0.01). No differences were detected between the rhG-CSF treatment group and the controls (P>0.05). The MAoP of the rats in the rhG-CSF treatment group (113.82±21.73 mmHg) was increased compared with that of the model group (P<0.05), and was restored to the level of the controls (P>0.05; Fig. 1E and F).

Histological examination indicated that medial hypertrophy of the pulmonary arteriole smooth muscle was evident in the model group (Fig. 1B) and that the WT of the model group was increased compared with that of the controls (31.74±3.09 versus 13.99±1.14%, P<0.01). The administration of rhG-CSF was shown to markedly attenuate the medial hypertrophy of the pulmonary arteriole smooth muscle (Fig. 1C), while the WT of the rhG-CSF treatment group (17.31±1.92%) was notably decreased compared with that of the model group (P<0.01; Fig. 1G).

The protective effects of rhG-CSF on the MCT-induced rat model of PH were markedly attenuated by the negative intervention of L-NAME. The RVSP of rats in the L-NAME intervention group (51.19±2.93 mmHg) was increased significantly compared with that of the rhG-CSF treatment group (P<0.01), while the MAoP was decreased (96.25±17.92 mmHg, P<0.01; Fig. 1E and F). Histological examination indicated that medial hypertrophy of the pulmonary arteriole smooth muscle was evident, similar to that observed in the model group (Fig. 1D), and the WT (31.97±3.22%) was increased compared with that of the rhG-CSF treatment group (P<0.01; Fig. 1G).

### EPC level in peripheral blood

**Figure 2:**
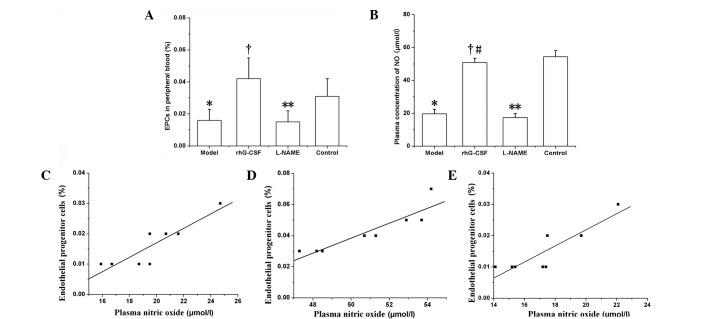
Levels of circulating endothelial progenitor cells (EPCs) and nitric oxide (NO). Data are presented as the mean ± standard deviation, n=8. (A) Numbers of EPCs in peripheral blood. ^*^P<0.01 compared with the control group; ^†^P<0.01 compared with the model and control groups; ^**^P<0.01 compared with the recombinant human granulocyte colony-stimulating factor (rhG-CSF) treatment group. (B) Plasma concentration of NO. ^*^P<0.01 compared with the control group; ^†^P<0.01 compared with the model group; ^#^P>0.05 compared with the control group; ^**^P<0.01 compared with the rhG-CSF treatment group. (C–E) Linear regression correlation between the number of circulating EPCs and the plasma concentration of NO in the (C) model (r=0.792, P<0.05); (D) rhG-CSF treatment (r=0.836, P<0.05) and (E) Nω-nitro-L-arginine methylester (L-NAME) intervention (r=0.889, P<0.05) groups.

The number of EPCs in the peripheral blood, assessed using flow cytometry, was significantly lower in the model group 21 days subsequent to the injection of MCT compared with the controls (0.016±0.007 versus 0.031±0.011%, P<0.01). The administration of rhG-CSF was shown to notably increase the number of EPCs (0.042±0.013%) compared with the numbers in the model and control groups (P<0.01). The intervention of L-NAME attenuated the effect of rhG-CSF on the circulating EPCs (0.015±0.007%, P<0.01; Fig. 2A).

### Plasma concentration of NO

The plasma concentration of NO was lower in the model group (19.66±2.78 μmol/l) than in the control group (54.31±3.81 μmol/l, P<0.01). The administration of rhG-CSF was shown to significantly increase the plasma concentration of NO compared with that in the model group (50.85±2.64 versus 19.66±2.78 μmol/l, P<0.01), and no differences in NO level were observed between the rhG-CSF treatment and control groups (P>0.05). The administration of L-NAME, which is an inhibitor NO synthase, suppressed the upregulation of plasma NO mediated by rhG-CSF (Fig. 2B). Furthermore, the plasma concentration of NO in each group was positively correlated with the number of circulating EPCs (P<0.05; Fig. 2C–E).

### EPC growth in vitro

**Figure 3:**
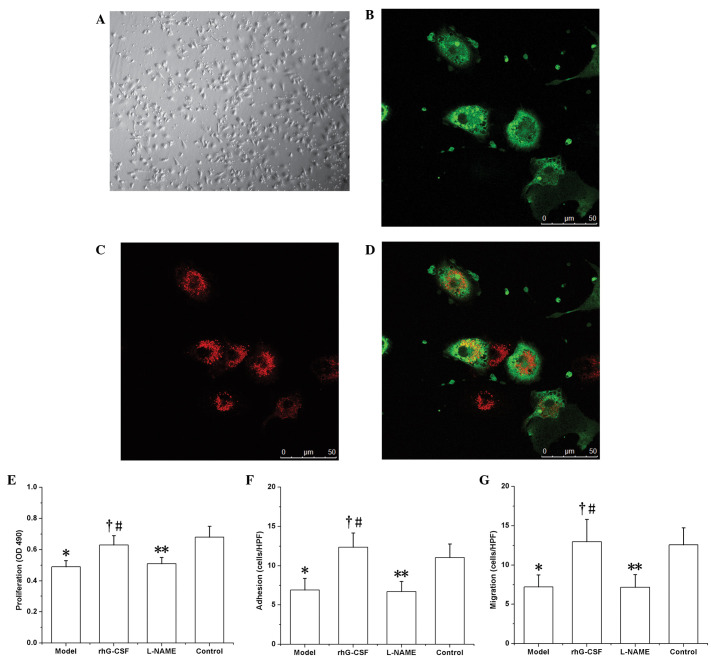
Function of circulating endothelial progenitor cells (EPCs) *in vitro*. (A–D) Identification of EPCs: EPCs from peripheral blood mononuclear cells of rats were cultured *in vitro* for seven days. (A) The cells showed a spindle-like morphology, the typical morphology of EPCs (magnification, ×100). The majority of the cells were immunopositive for (B) fluorescein isothiocyanate-conjugated lectin from Ulex europeus agglutinin-1 (FITC-UEA-1) and (C) DiI-labeled acetylated low-density lipoprotein (DiI-acLDL). The cells positive for the two labels (see yellow fluorescence) were recognized as EPCs in differentiation (D). (E–G) EPC functions i*n vitro*: (E) Proliferation, (F) adhesion and (G) migration. Data are presented as the mean ± standard deviation, n=8. ^*^P<0.05 compared with the control group; ^†^P<0.05 compared with the model group; ^#^P>0.05 compared with the control group; ^**^P<0.01 compared with the recombinant human granulocyte colony-stimulating factor (rhG-CSF treatment group).

Following seven days of culture, abundant spindle-like cells were observed to be adhered to the bottom of the culture plate (Fig. 3A). Subsequent to binding with FITC-UEA-1, these cells exhibited green fluorescence (Fig. 3B), and red fluorescence was exhibited following phagocytosis of DiI-acLDL (Fig. 3C). The cells positive for the two labels under laser scanning confocal microscopy (observed as yellow fluorescence) were recognized as EPCs undergoing differentiation (Fig. 3D).

### EPC function in vitro

The functions of the EPCs were all downregulated in the model group compared with those of the controls. In the test of proliferation ability *in vitro*, the absorbance (at 490 nm; OD) of the formazan supernatant (dissolved in DMSO) was 0.49±0.04 in the model group, which was decreased compared with that of the control group (0.68±0.07, P<0.01). Similar results were observed with regard to adhesion ability (6.93±1.47 cells/HPF in the model group versus 11.05±1.73 cells/HPF in the control group, P<0.01) and migratory ability (7.22±1.53 cells/HPF in the model group versus 12.58±2.15 cells/HPF in the control group, P<0.01). In the rhG-CSF treatment group, these functional indices were all upregulated compared with those of the model group (proliferation, OD 0.63±0.06; adhesion, 12.35±1.82 cells/HPF; migration, 12.97±2.84 cells/HPF; P<0.01; Fig. 3F–H). Consistent with the effect of L-NAME on the number of EPCs in the peripheral blood, the functions of the EPCs in the L-NAME intervention group, i.e. proliferation (OD 0.51±0.04), adhesion (6.73±1.28 cells/HPF) and migration (7.18±1.62 cells/HPF) were all downregulated compared with those of the rhG-CSF treatment group (P<0.01; Fig. 3E–G).

## Discussion

In the present study, it was demonstrated that in a rat model of PH, the number and function of circulating EPCs were markedly decreased. Moreover, there was a downregulation in the plasma concentration of NO, which was positively correlated with the number of circulating EPCs. Administration of rhG-CSF elevated the plasma level of NO, upregulated the number and function of circulating EPCs and effectively improved pulmonary hemodynamics and vascular reconstruction. Furthermore, the positive correlation between the concentration of plasma NO and circulating EPCs was also observed in the rhG-CSF treatment group. However, the protective effects of rhG-CSF on PH were impaired by the L-NAME-mediated downregulation of NO and the EPCs. These results indicate that rhG-CSF prevents or attenuates the progression of MCT-induced PH by improving vascular injury repair mechanisms via the NO-mediated upregulation of EPCs.

PH is characterized by the persistent contraction and medial hypertrophy of extensive pulmonary arterioles, which may thus result in the abnormal elevation of pulmonary artery pressure. Despite the fact that drug interference may improve the symptoms and decrease the occurrence of heart attack, the effects are partial and limited and right ventricular failure or mortality is likely be the fate of numerous patients in the clinic. It is therefore crucial to prevent the progression of the pathological changes in the initial stages of this disease. Studies have shown that the early pathological change in PH is the injury of the arteriolar endothelium, resulting from excessive apoptosis of the endothelial cells. This then induces vasodilatation dysfunction, the over-proliferation of vascular smooth muscle cells and fibroblasts, ultimately resulting in the occurrence of PH (19–21). Thus, reconditioning the injured endothelium as early as possible may prevent this course of the disease.

EPCs, which are co-precursors with hematopoietic stem cells, have been suggested to be pivotal to the homeostasis and repair of the vascular endothelium. Studies have shown that the number of circulating EPCs was decreased in patients with PH (5,6) and that PH was alleviated by the transplantation of exogenous EPCs in an experimental animal model (22,23), which suggested that the impaired condition of the EPCs contributed to the occurrence of PH. An appropriate strategy to upregulate EPCs may be used to prevent this course effectively. In the present study, the changes in pulmonary hemodynamics and histology that were evident following the injection of MCT in SD rats appeared consistent with those in patients with PH and were suited to performing further observations on circulating EPCs. It was observed that the number of EPCs in the peripheral blood decreased significantly in the PH models compared with the number in the controls. In addition, when cultured *in vitro*, the functions of EPCs, such as proliferation, adhesion and migration, were downregulated. It is possible that the repair processes secondary to the ongoing lesion of the vascular endothelium may lead to the large consumption of circulating EPCs and potentially exhaustion. Bone marrow EPCs and precursors, as the reservoir of circulating EPCs, may also be impaired, in number and function (24,25). A reduction in the protective mechanism of EPCs therefore exacerbates vascular damage and dysfunction.

Data have shown that G-CSF is able to increase the number of circulating EPCs, upregulate the maturation and proliferation capacity of EPCs (26) and then accelerate the repair of the injured vessel and myocardium. Furthermore, G-CSF has exhibited a protective effect in cardiovascular diseases (27). The mobilization of bone marrow stem cells using G-CSF has also been shown to effectively to prevent the progression of PH (9). In the present study, it was demonstrated that, following the administration of rhG-CSF, the number of EPCs in the peripheral blood was increased significantly and the function of the EPCs was upregulated. As a result, there was an improvement in the pathological changes in the pulmonary artery, in addition to an alleviation of pulmonary artery pressure.

To further investigate the possible mechanism underlying the protective effect of G-CSF in PH, we measured the plasma concentration of NO in each group. It was shown that the level of plasma NO decreased significantly in the rat model of PH compared with the controls, and that the administration of rhG-CSF effectively induced the upregulation of NO and accelerated the repair of the injured pulmonary artery endothelium by mobilizing the bone marrow EPCs (10), while reducing the apoptosis of EPCs (11). Furthermore, the level of NO was demonstrated to be positively correlated with the number of circulating EPCs in the PH model and rhG-CSF treatment groups. However, the exact signaling pathway involved in the G-CSF-induced upregulation of NO and EPCs was not elucidated in the current study. The study by Ueda *et al*(14) demonstrated that G-CSF phosphorylated and activated endothelial NO synthase (NOS) in the acute stage of myocardial infarction and increased NO production, thus inducing protective effects on the myocardium. Our observation regarding the change in plasma NO levels in PH was consistent with that of the study by Ueda *et al*, which suggests that the same signaling pathway may be involved. Furthermore, the administration of L-NAME, which acted as an NOS inhibitor in the present study, was accompanied by reductions in the plasma concentration of NO and in the number and function of circulating EPCs, thus attenuating the protective effect of rhG-CSF in PH. This further demonstrated the protective effects of the NO-mediated upregulation of EPCs in PH.

In conclusion, the current study indicated that the administration of rhG-CSF may represent a novel strategy for the treatment of PH. The treatment may effectively prevent the disorder by upregulating the number and function of circulating EPCs via the NO system, and then accelerate the reparation of the pulmonary artery endothelial lesion. However, the prospective efficacy and side-effects of rhG-CSF remain important issues to be investigated in advanced experimental and *in vivo* studies.
